# Implication of integrin α2β1 in senescence of SK-Mel-147 human melanoma cells

**DOI:** 10.18632/aging.203309

**Published:** 2021-07-13

**Authors:** Nadezhda I. Kozlova, Galina E. Morozevich, Albert E. Berman

**Affiliations:** 1 VN Orekhovich Institute of Biomedical Chemistry, Moscow 119121, Russia

**Keywords:** tumor progression, senescence, integrins, signaling, Akt isoforms

## Abstract

This investigation addressed the impact of integrin-initiated signaling pathways on senescence of tumor cells. In a model of human SK-Mel-147 melanoma cells, the silencing of integrin α2β1 strongly reduced cell proliferation and enhanced the percentage of SA-β-Gal-positive cells, a phenotypic feature of cellular senescence. These changes were accompanied by a significant increase in the activity of Akt and mTOR protein kinases and also in the expression of p53 and p21 oncosuppressors. Pharmacological inhibition of Akt and mTORC1 and genetic inhibition of p53 and p21 reduced the senescence of α2β1-depleted SK-Mel-147 cells to the level of control cells. Based on our earlier data on the non-canonical functions of Akt isomers in the invasion and anoikis of SK-Mel-147 cells, we investigated the role of Akt isomers in senescence induced by α2β1 suppression. The inhibition of Akt1 strongly reduced the percentage of SA-β-Gal-positive cells in the α2β1-depleted cell population, while the inhibition of Akt2 did not have a noticeable effect. Our data demonstrated for the first time that α2β1 is involved in the protection of tumor cells against senescence and that senescence, which is induced by the downregulation of α2β, is based on a signaling mechanism in which Akt1 performs a non-canonical function.

## INTRODUCTION

Cellular senescence involves a complex number of intracellular processes that lead to an irreversible arrest of cell growth. The phenomenon of replicative senescence is well known and is induced by the shortening of telomeric DNA after each replicative cycle [1]. In addition, cellular senescence can also be induced by various stress signals, including reactive oxygen species (ROS), DNA damage, lack of growth factors, etc [2, 3]. The discovery of oncogene-induced senescence, which is caused by the activation of mutated oncogenes, has been of great importance in understanding the mechanisms of tumor growth. In normal proliferating cells, this process is the most important safeguard that prevents tumor development, while during oncogenic transformation, specific mechanisms have evolved to counteract the oncogene-induced proliferation [4, 5].

Numerous studies indicate that cellular senescence is controlled by intra- and extracellular signals, including signals generated during cell adhesion to the extracellular matrix (ECM) [6–8]. Binding of cells to the matrix results from the interaction of cell surface transmembrane receptors (integrins) with ECM proteins. Integrins represent a large family (24 members) of heterodimeric proteins that are characterized by great structural similarities [9, 10]. Integrins play a key role in diverse physiologic and pathological conditions [10–12].

The implications of these receptors in oncogenic transformation and tumor progression have been documented by a large number of studies [13, 14]. However, their involvement in cellular senescence and also the mechanisms acquired by tumor cells for overcoming this process remain poorly studied. Researchers have only characterized a few of the most common integrins to some extent. Downregulation of the laminin-specific receptor α6β4 significantly inhibited senescence and decreased the expression of p53 tumor suppressors in umbilical endothelial cells, indicating a stimulating effect of α6β4 on the senescence of these cells [15]. Stimulation of cellular senescence by α6β4 integrin was confirmed in a study of several lines of tumor cells subjected to IR radiation, which showed that α6β4-induced signals switch the response of the irradiated cells from apoptosis to senescence [16]. In contrast, in airway epithelium, downregulation of a6b4 caused a 10-fold increase in the number of cells displaying a senescence phenotype [17].

The contribution of the β3 integrin family to the mechanisms that regulate cellular senescence is also poorly studied. In glioblastoma cells, β3 signaling is critical for resisting senescence [18], while in primary breast fibroblasts and breast carcinoma cells, β3-integrin overexpression stimulates senescence [19].

Notably, in the integrin family, β1 receptors have been the least explored for their impact on cellular senescence despite being the most common receptor in the animal kingdom. In particular, no published data are available about the effects of integrins α2β1 and α3β1 on senescence mechanisms.

Mitogen-activated protein kinases (MAPK) plays an important role in integrin-dependent mechanisms of tumor growth, of which the extracellular signal-related kinase (Erk), the Jun aminoterminal kinase (JNK), and p38-MAPK have been studied in more detail [20–22] These molecules are involved in signaling cascades that can induce various, often opposite, responses in different cell types [23]. In human lung cancer cells and mice xenograft models, integrin αvβ6 has been shown to promote cell proliferation, invasion and tumor growth, and suppress apoptosis and senescence through upregulation of Erk signaling [24]. In contrast, in prostate cancer cells, integrin α2β1 reduced proliferation due to the activation of protein kinase P38-MAPK [25]. This finding is in line with our results showing that integrin α2β1 is involved in the protection of breast carcinoma cells from anchorage-dependent apoptosis (anoikis) through a mechanism based on the inhibition of Erk signaling [26]. The role of Erk signaling in cellular senescence has been documented in a number of publications which indicate that Erk can mediate both stimulation and inhibition of senescence. In glioma cells, inactivation of the Ras/Erk pathway strongly stimulated senescence and apoptosis indicating that Erk can mediate signals that suppress senescence and apoptosis [27]. On the other hand, a number of studies suggest that aberrant Erk activation can promote cell death and senescence [23]. Yet, despite significant progress in elucidating the contribution of MAP kinases to cellular senescence, their specific role in integrin-initiated pathways affecting this process is not well understood.

In our recent publications, a non-canonical function of the Akt protein kinase, a key mediator in many signaling cascades, was documented in the transduction of signals initiated by α2β1, α3β1, and α5β1 in a highly invasive line of human melanoma cells [28–30]. The stimulation of invasion and the process of overcoming anoikis, which are demonstrated by α2β1, α3β1, and α5β1occur through a mechanism based on inhibiting the activity of one of the Akt isoforms that exhibits functions uncharacteristic (non-canonical) of this protein kinase.

The aim of this investigation was to determine whether integrin α2β1 influences the signaling mechanisms that control tumor cell senescence and also to identify the role of Akt isomers in its signaling. In a model of a human melanoma cell line, it was demonstrated for the first time that α2β1 is involved in rescuing tumor cells from senescence; its impact was mediated by a signaling mechanism based on the non-canonic activity of Akt1 kinase.

## RESULTS AND DISCUSSION

### Downregulation of integrin α2β1 enhances the cellular senescence of SK-Mel-147 human melanoma cells

**Figure 1:**
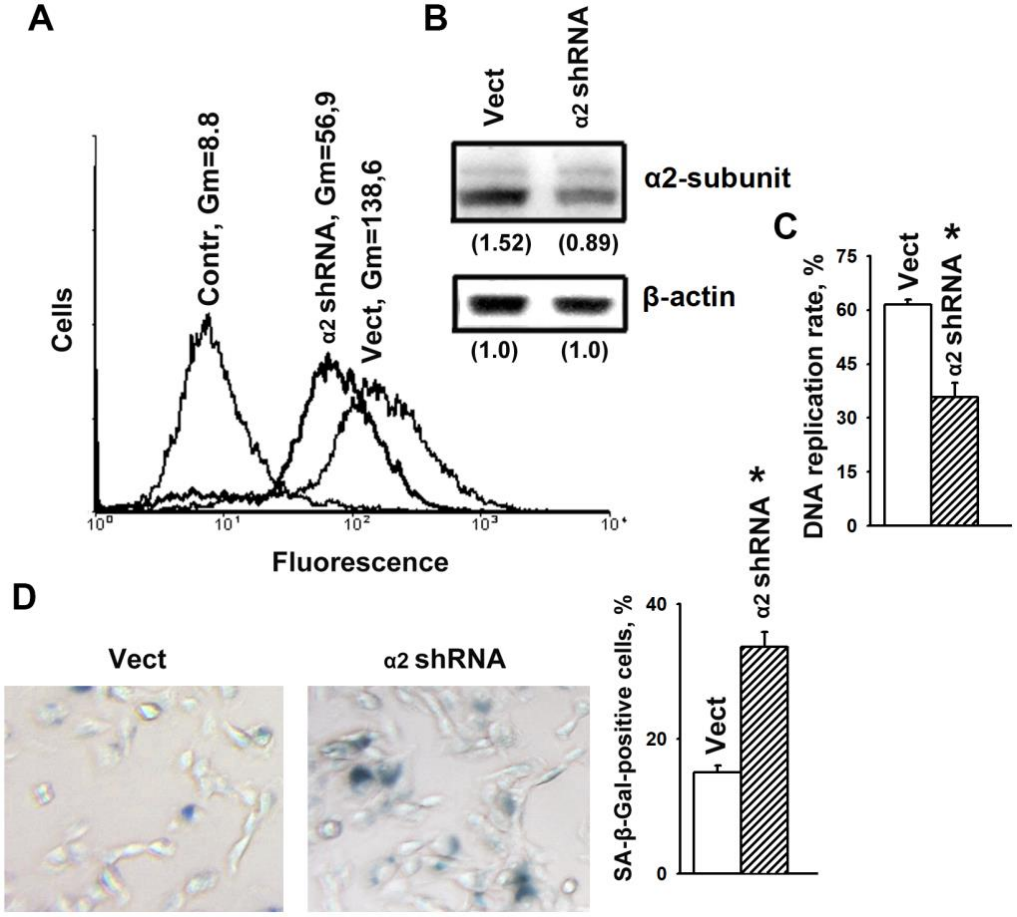
**α2β1 deficiency caused senescence of SK-Mel-147 melanoma cells.** The cells were transduced with the lentiviral plasmid vector pLKO.1-puro containing α2 shRNA or scramble shRNA (Vect) and selected using puromycin. (**A**) FACS analysis of α2β1 cell surface expression. Contr: scramble shRNA transduced cells were stained with FITC-conjugated anti-mouse IgG; Vect: scramble shRNA transduced cells were probed with α2β1 mAb and FITC-conjugated anti-mouse IgG; α2 shRNA: α2 shRNA transduced cells were probed with α2β1 mAb and FITC-conjugated anti-mouse IgG; Gm, geometric mean fluorescence intensity. Shown are representative FACS histograms. (**B**) Western-blotting of the cellular lysate proteins. Cell lysate proteins were run on SDS-PAGE as described in Materials and Methods. The blots were probed with 1:1000 dilution of antibodies to the specified proteins and treated as described in Materials and Methods. Numbers below the bands indicate the α2 band densities normalized against β-actin. Shown are representative blots. (**C**) α2β1 downregulation reduced the rate of DNA replication of SK-Mel-147 cells. The cells were transduced with scramble shRNA or α2 shRNA and treated as described in Materials and Methods. DNA replication is presented as the ratio (%) Alexa-488-stained cells/Hoechst-stained cells. The results of three independent experiments are shown (M ± SEM); *, ρ < 0.05. (**D**) SA-β-Gal staining. Cells staining positive for SA-β-Gal showed blue color sedimentation; magnification: × 200. Quantification was performed by counting 200 SA-β-Gal-positive cells in duplicate plates and is presented as a percent of total cells. The results of three independent experiments are shown (M ± SEM); *, ρ <0.05.

To determine the effects of α2β1 upon the mechanisms of senescence, we assessed the effect of its downregulation on the activity of beta-galactosidase, a typical marker of senescence. Suppression of this integrin was carried out by transducing the cells with a plasmid clone expressing α2-specific shRNA. Western blot and flow cytometry data showed high efficiency of the used clone (Figure 1A, 1B). As shown in Figure 1C, 1D, blocking the expression of α2β1 led to a sharp increase in cellular senescence, as well as a significant decrease in the proliferation of the cells. Inhibition of proliferative activity is a typical trait of cellular senescence.

We have previously shown that knockdown of α2β1 was accompanied by a significant increase in anoikis and in the levels of p53 and p21 proteins in SK-Mel-147 cells [28]. Numerous studies have shown that the oncosuppressor p53 and its downstream target p21 play a key role in the mechanism of cellular senescence and the choice of a cell to respond with either irreversible growth arrest or apoptosis [2]. Decreased proliferation of α2β1-depleted cells (Figure 1C) might be the result of increased activity of p53/p21 that induces both senescence and cell death.

### The role of p53/p21 signaling in SK-Mel-147 cell senescence induced by α2β1 integrin downregulation

The p53 tumor suppressor and its downstream effector CDK inhibitor p21 play an essential role in the mechanisms of cell proliferation, apoptosis, and senescence [2, 31]. It has been shown that the initial phase of senescence depends on the high levels of p53 and p21, which then decreases [2, 32] Of importance, the choice of cellular response to a stress stimulus between apoptosis and senescence depends on the level and duration of the stress signal; a weak and transient signal leads to a transient arrest of growth, while senescence requires a stable signal. However, exceeding the threshold level of a stress stimulus leads to cell death [2, 33]. The choice between apoptosis and senescence is also determined by the ratio between p53 and p21; the stimulating effect of p21 on senescence is based on its ability to inhibit apoptosis, while the increased activity of p53 inhibits p21 [2].

**Figure 2:**
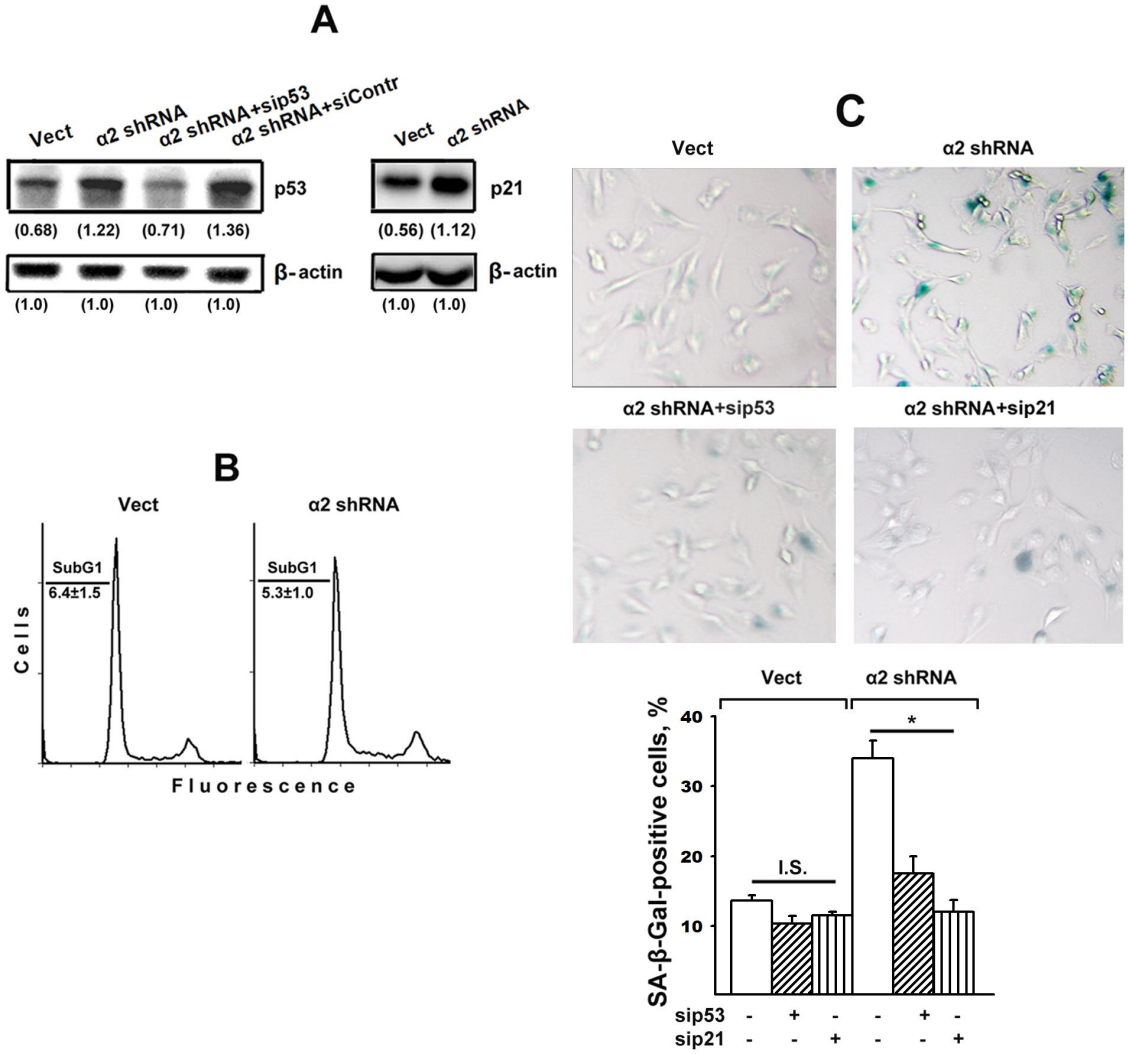
**Silencing of p53 and p21 reversed the stimulatory effect of α2β1 knockdown on senescence of SK-Mel-147 melanoma cells.** The cells were transduced with the appropriate vectors and transfected with p53- or p21-specific siRNAs as described in Materials and Methods. (**A**) Western-blotting of the cellular lysate proteins. The procedures were performed as described in Materials and Methods and the legend to Figure 1. Numbers below the bands indicate the p53 or p21 band densities normalized against β-actin. Shown are representative blots. (**B**) Apoptosis assay. Cells transduced with the appropriate vectors were stained with propidium iodide, analyzed by flow cytometry (Materials and Methods) and the percentage of cells with subG1 DNA was determined. Shown are representative histograms; SubG1 values were derived from three independent experiments (M ± SEM). (**C**) SA-β-Gal staining. The cells were treated and counted as described in Materials and Methods and in the legend to Figure 1; magnification: × 200. The results of three independent experiments are shown (M ± SEM); *, ρ < 0.05. Vect, scramble shRNA transduced cells; α2 shRNA, α2 shRNA transduced cells; I.S, insignificant.

Figure 2A shows that α2β1 downregulation was accompanied by a two-fold increase in the expression of p53 and p21 proteins, which is in agreement with our earlier data [28]. Based on this result and the previous citations, it may be inferred that the stress signal induced by downregulating α2β1 in studied cells led to apoptosis and/or senescence. One of the phenotypic features of apoptosis is the accumulation of a subpopulation of cells with low (subG1) DNA content in its total cell population. The data (Figure 2B) indicate that the percentage of subG1 cells in the control SK-Mel-147 population did not exceed 5–7% and did not change in the population with reduced α2β1 expression after 48 hours of growth in culture media. Thus, the increased levels of p53/p21 did not lead to increased apoptotic death of the studied cells.

It could be assumed that the effect of a2β1 downregulation on senescence was not due to increased p53/p21 signaling but instead was initiated by another signaling pathway, such as p16/Rb. To clarify this issue, we investigated the effect of blocking the expression of each of these proteins on the senescence of SK-Mel-147 cells with reduced α2β1 expression. As seen in Figure 2A, in cells lacking α2β1, siRNA-induced silencing of p53 reduced the expression of this protein to the level of control cells and, as evidenced in Figure 2C, suppression of p53 and p21 sharply reduced the ratio of senescent cells in the α2β1-downregulated population. In cells with intact α2β1, the effect of sip53 and sip21 was insignificant most likely because of already low senescence of these cells.

Thus, the deprivation of melanoma cells of one of the β1-family receptors is a stress factor that induces the senescence phenomenon by activating the p53/p21 signaling pathway.

### The role of Erk signaling in SK-Mel-147 cell senescence induced by downregulation of α2β1

**Figure 3:**
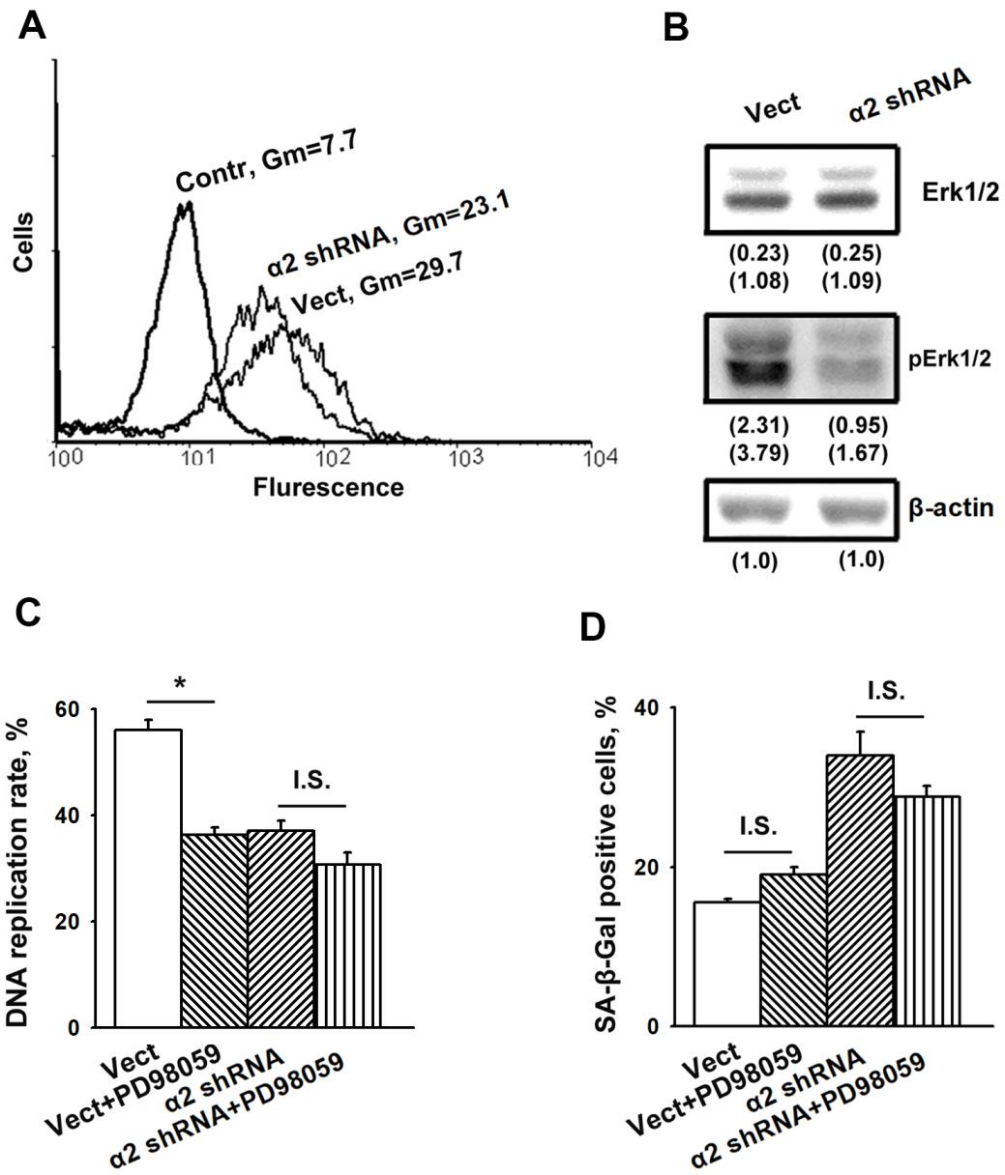
**Inhibition of Erk protein kinase did not affect senescence of SK-Mel-147 melanoma cells.** (**A**) FACS analysis of pErk expression. Cells transduced with the appropriate vectors were permeabilized using 100% methanol, probed with pErk mAb and FITC-conjugated anti-mouse IgG according to the manufacturer's protocol (Cell Signaling Tech, USA). Other designations are the same as in Figure 1. (**B**) Western-blotting of the cellular lysate proteins. The procedures were performed as described in Materials and Methods and the legend to Figure 1. Numbers below the bands indicate the protein band densities normalized against β-actin. Shown are representative blots. (**C**) Effect of Erk inhibition on the rate of DNA replication of SK-Mel-147 cells. Cells transduced with the appropriate vectors were incubated in serum-reduced medium, containing 25 μM Erk inhibitor PD98059 for 24 h, and treated as described in Materials and Methods. DNA replication was calculated as described in the Legend to Figure 1. The results of three independent experiments are shown (M ± SEM); *, ρ < 0.02; I.S., insignificant. (**D**) Effect of Erk inhibition on senescence of SK-Mel-147 cells depleted of α2β1. Cells transduced with the appropriate vectors were incubated overnight in serum-reduced medium, containing 25 μM Erk inhibitor PD98059 followed by SA-β-Gal staining. The results of three independent experiments are shown (M ± SEM). Vect, scramble shRNA transduced cells; α2 shRNA, α2 shRNA transduced cells. I.S., insignificant.

As noted above, the information on the contribution of MAPK to integrin-dependent signaling mechanisms that control cellular senescence is limited. Thus, it seemed to be of interest to determine the implication of Erk signaling in demonstrated in this study modifications of proliferation and senescence induced by α2β1 suppression. Figures 3A, 3B show that blocking α2β1 significantly reduced the active (phosphorylated) form of Erk in SK-Mel-147 cells. This result may indicate that in the studied cells, Erk signaling is involved in the mechanism of proliferation but not senescence. To verify this suggestion, we investigated the effect of PD98059, a specific inhibitor of MEK/Erk, on proliferation and senescence of the control and α2β1-depleted SK-Mel-147 cells. A 24 h treatment with the Erk inhibitor decreased DNA replication rates of control cells by approximately 30% (Figure 3C), whereas DNA replication of the α2β1-depleted cells was unaffected, most likely because it had already been highly reduced. On the other hand, inhibition of Erk did not affect the proportion of SA-β-Gal positive cells in the populations of control and α2β1-depleted cells (Figure 3D). These results suggest that increase in senescence caused by depletion of α2β1 in melanoma cells is not due to the reduction in Erk activity.

### The role of the PI3K/Akt/mTOR pathway in SK-Mel-147 cell senescence induced by downregulation of α2β1

One of the most studied and important signaling mechanisms underlying oncogene-induced senescence is the PI3K/Akt/mTOR pathway [34, 21]. The critical point of this pathway is transmitting the activating signals from Akt through TSC2 to the mTORC1 complex, followed by activation of p53/p21 signaling [34]. This pathway is typical for cell responses to stress challenges, such as DNA damage, oxidative stress, radiation exposure, etc. Under non-stress conditions, Akt performs a well-known (canonical) function aimed at stimulating cell proliferation, tumor progression, overcoming apoptosis, etc., which is realized through the activation of a number of transcription factors, such as NF-kB, cMyc, etc. [35]. A protective function of Akt against cell senescence (particularly in maintaining the integrity of telomeres by the phosphorylation of hTERT and its translocation from the cytoplasm to the nucleus) has previously been described [36, 37]. RASSF1-mediated activation of p21 led to senescence of lung adenocarcinoma cells through the inhibition of Akt [38].

Our recent studies have shown that in SK-Mel-147 cells depleted of integrins α2β1, α3β1, and α5β1, Akt-mediated signals suppressed an *in vitro* cell invasion and enhanced anoikis (i.e. exhibited non-canonical properties) [28–30]. In this study, we assessed the implication of Akt signaling in senescence of SK-Mel-147 cells caused by the suppression of integrin α2β1.

**Figure 4:**
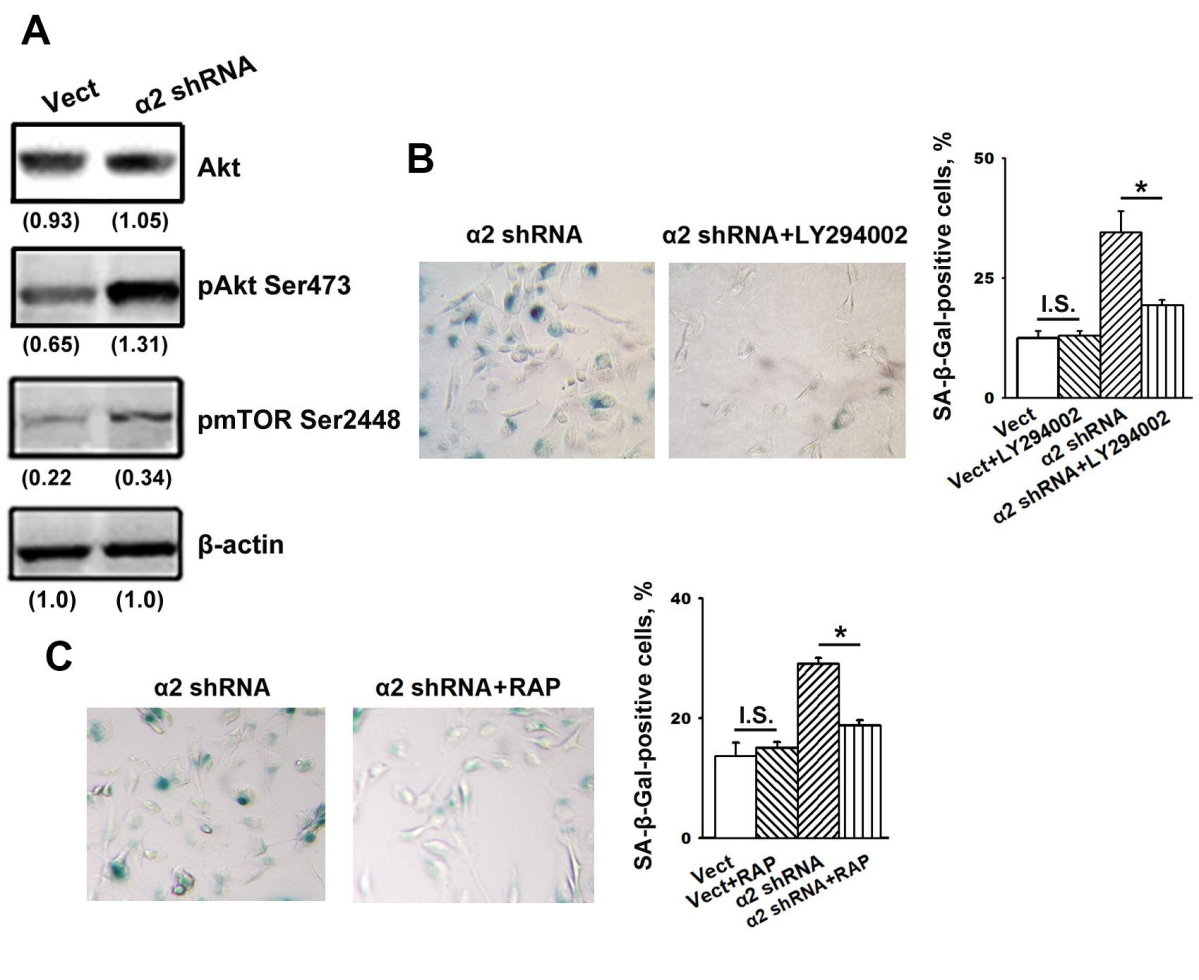
**Inhibition of Akt and mTOR protein kinases reversed the stimulatory effect of α2β1 knockdown on senescence of SK-Mel-147 cells.** (**A**) Western-blotting of the cellular lysate proteins. The procedures were performed as described in Materials and Methods and the legend to Figure 1. Numbers below the bands indicate the protein band densities normalized against β-actin. Shown are representative blots. (**B**) Effect of PI3K/Akt inhibitor LY294002 on senescence of SK-Mel-147 cells depleted of α2β1. Cells transduced with the appropriate vectors were incubated overnight in serum-reduced medium, containing 25 μM PI3K/Akt inhibitor LY294002 followed by SA-β-Gal staining; magnification: × 200. Shown are the results of three independent experiments (M ± SEM). ρ < 0.02; I.S., insignificant. (**C**) Effect of mTORC1 inhibitor Rapamycin on senescence of SK-Mel-147 cells depleted of α2β1. Cells transduced with the appropriate vectors were incubated overnight in serum-reduced medium containing 200 nM Rapamycin followed by staining for β-Gal. Shown are the results of three independent experiments (M ± SEM). Vect, scramble shRNA transduced cells; α2 shRNA, α2 shRNA transduced cells; RAP, Rapamycin. *, ρ < 0.05; I.S., insignificant.

The data presented in Figure 4A show that blocking α2β1 led to a sharp increase in the phosphorylated (active) form of Akt (pAkt) with no effects on Akt total protein levels and also enhanced the expression of the Akt downstream effector mTOR protein kinase.

We suggested that enhanced activity of these protein kinases is not just a trait accompanying increased senescence but rather can be attributed to their involvement in the mechanisms of senescence. To verify this suggestion, we investigated the effect of inhibitors of the Akt/mTOR pathway on senescence in melanoma cells depleted of α2β1. To block this pathway, the cells were treated with a PI3K inhibitor (LY294002) and an mTORC1 inhibitor (rapamycin). As shown in Figure 4B, 4C, the suppression of PI3K/Akt/mTOR signaling significantly attenuated senescence induced by α2β1 knockdown in SK-Mel-147 cells. Thus, signals transmitted by PI3K/Akt/mTOR play an important role in senescence induced by α2β1 integrin deprivation.

### The role of Akt isozymes in SK-Mel-147 cell senescence induced by α2β1 integrin knockdown

In our studies [27, 29], we showed that the non-canonical effect of Akt-induced signals on the invasion and anoikis of SK-Mel-147 cells deficient in α2β1 was due to the activity of the Akt1 isozyme, while other Akt isoforms did not exhibit non-canonical properties. In the present investigation, we attempted to determine the function of Akt isoforms in senescence of these cells.

**Figure 5:**
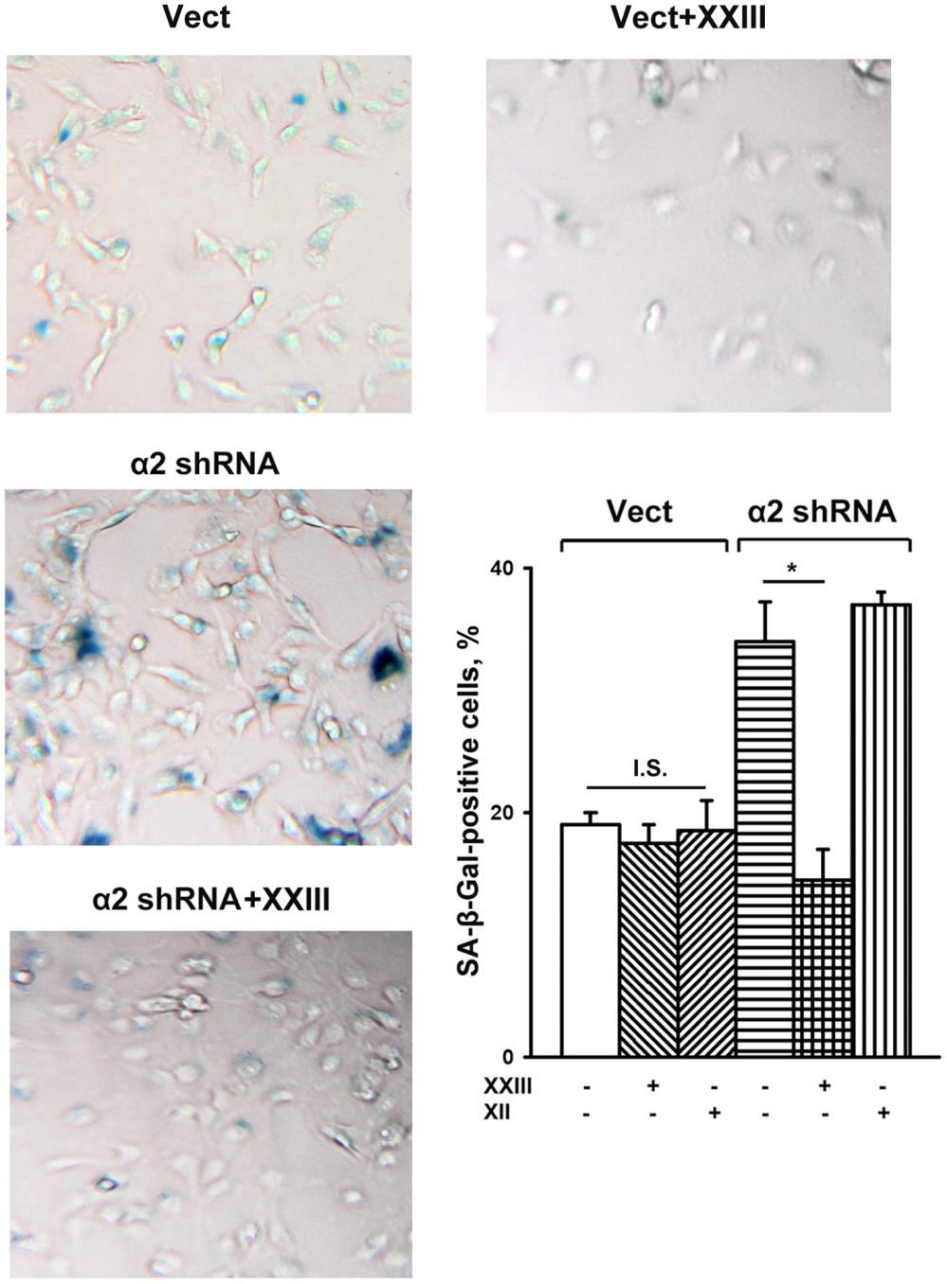
**Effect of Akt isoform inhibitors on senescence of SK-Mel-147 cells.** The cells were transduced with the appropriate vectors as described in Materials and Methods, treated for 24 h at 37° C with 3 μM Akt1-specific inhibitor XXIII or 5μM Akt2-specific inhibitor XII followed by SA-β-Gal staining; magnification: × 200. Shown are the results of three independent experiments (M ± SEM). Vect, scramble shRNA transduced cells; α2 shRNA, α2 shRNA transduced cells;. *, ρ < 0.05; I.S., insignificant. The SA-β-Gal staining data presented in Figure 1D and Figure 5 were obtained in the same series of experiments.

To this end, we investigated the effect of specific inhibitors of individual Akt isoforms on senescence of control and a2β1-depleted SK-Mel-147 cells. Figure 5 shows that Akt1- and Akt2-specific inhibitors had no significant effect on the senescence of melanoma cells that sustained a high level of α2β1 expression. In cells depleted of α2β1, the Akt1-specific inhibitor reduced the level of the SA-β-Gal-positive population by about 50%, while inhibition of the Akt2 isoform did not affect senescence.

As noted above, the data on the role of integrins in the development of senescence are contradictory, as evidenced by the fact that the same receptor can both stimulate and suppress the development of the senescent phenotype [15–17]. Signaling mechanisms underlying these opposite cellular reactions are poorly studied. Therefore, it is of interest to compare our results on the role of the Akt-mediated pathway in melanoma cell senescence caused by suppressing α2β1 signaling with the observation that the same pathway transmits signals stimulating lung carcinoma cell senescence caused by radiation-induced activation of α6β4 integrin signaling [16]. It is plausible that this difference is due to the strength of a stress signal produced in one case by the suppression of integrin and in the other by the irradiation of cells. This assumption is supported by the authors’ observation that blocking α6β4 switches the path of the cells from senescence to apoptosis. One would suggest that both α6β4 and α2β1 play a role in protecting cells against a worse scenario; in lung carcinoma cells exposed to radiation, α6β4 protects against apoptosis, while in melanoma cells, α2β1 protects against oncogene-induced senescence.

The involvement of individual Akt isoforms in the mechanisms of cellular senescence is also largely unexplored. Of the three Akt isoforms, only Ak1 knockdown led to damage of telomeres in fibrosarcoma cells, which indicates that the function of this kinase is to override senescence [36].

Akt1-null mouse embryonic fibroblasts appeared more sensitive to UV-induced senescence than Akt2-null cells [39]. These findings indicate that, in different cells, the role of Akt1 in senescence complies with its canonical functions, which are to promote cell proliferation and survival, tumor metastasis, protection against apoptosis, etc. However, as shown in this investigation, in melanoma cells lacking α2β1, the Akt1 isoform performs an opposite, non-canonical function. This observation is consistent with our earlier results on the non-canonical activity of Akt1 that were obtained when studying its effect on anoikis and the invasive activity of SK-Mel-147 cells depleted of α2β1, α3β1, and α5β1 integrins [28–30]. We suggest that knockdown of α2β1 is a stress factor that induces the senescence phenotype in SK-Mel-147 cells via the RAS/PI3K/Akt1/mTORC1/p53/p21 pathway, resulting in a decrease in proliferation, invasive activity, and resistance to anoikis of the cells.

A consequence of the non-canonical activity of Akt is that its inhibition did not lead to the death of tumor cells but instead restored their invasive activity [29]. The non-canonical Akt activity raises the question of the risk of using this and other protein kinases as targets in targeted cancer therapies given that anti-tumor therapy that is based on the suppression of protein kinases (including Akt) is currently broadly employed [40]. Further progress in this field will require more advances in studies devoted to the mechanisms that link cell surface receptors (including integrins) and signaling pathways in various types of tumor cells.

## MATERIALS AND METHODS

### Cell culture and chemicals

SK-Mel-147 human melanoma line was obtained from the Memorial Sloan Kettering Cancer Center (USA). Cells were cultured in DMEM medium containing 10% fetal calf serum, 2 mM L-glutamine, 100 U/ml penicillin, and 100 μg/ml streptomycin and incubated at 37° C in an atmosphere with 5% CO_2_. p53- and p21-specific siRNA were from Santa Cruz Biotech (USA) Polyclonal antibodies against α2-integrin subunit and monoclonal antibodies to the integrin α2β1 were, respectively, from Chemicon and BD PharMingen (USA). Polyclonal antibodies against protein kinases Akt and Erk and their phosphorylated forms (pAkt Ser473 and pErk Thr202/Tyr204), phospho-mTOR (pmTOR Ser2448), and proteins p53, p21 were from Cell Signaling Tech (USA). PI3K/Akt inhibitor, LY294002; Akt1 inhibitor, XXIII; Akt2 inhibitor, XII; ERK inhibitor, PD98059, and mTOR inhibitor, Rapamycin, were from Calbiochem, and other chemicals were from Sigma.

### Transduction of cells with shRNA

Bacterial glycerol clones containing lentiviral plasmid vector pLKO.1-puro with shRNA specific for the α2-integrin subunit and pLKO.1-puro lentiviral vector with scramble shRNA were purchased from Sigma. Lentivirus was produced in HEK293T cells by co-transfection with shRNA-containing or scrambled shRNA-containing vectors with packing plasmids as described earlier [41]. Cells were transduced with lentivirus in the presence of polybrene (8 μg/ml) and selected with puromycin (1–2 μg/ml) for 2–4 days.

### siRNA transfection

siRNA duplexes, both control and specific to the p-53 and p-21 proteins, as well as siRNA transfection reagent were from Santa Cruz Biotech. (CA, USA). The procedure was performed as described earlier [42]. Briefly, 1–2 x 10^5^ cells in 1 ml of antibiotic-free DMEM + 10% FBS were plated into each well of 6-well culture plates. After reaching ~50% confluence the cells were transfected with 50 nM siRNA (final concentration) for 70 h. Then cells were harvested with trypsin/EDTA, washed and used for subsequent experiments.

### Flow cytometry

1x10^5^ cells were fixed with 70% ethanol, rinsed in PBS, resuspended in 1 ml citrate buffer containing 50 μg/ml propidium iodide and 10 μg/ml RNAse A followed by storing on ice for 3 h. Cell surface expression of α2β1 integrin was assessed by treating the cells with anti-α2β1 (BD PharMingen), followed by staining with FITC-conjugated secondary antibody and fixation with 2% formaldehyde. The cells were analyzed using Becton Dickinson flow cytometer.

### Senescence assay

Cells were plated in 12-well plates for 24 h, rinsed in PBS, fixed and incubated overnight at 37° C with the staining solution containing the X-Gal substrate (BioVision, USA). The development of blue color was detected visually under the microscope and the cells staining positive for SA-β-Gal were counted as a percentage of the total cell number. The images were captured and processed using Adobe Photoshop CS5.

### DNA replication

DNA replication was assayed by incorporation of EdU (5-ethynyl-2′-deoxyuridine) followed by staining of replicating and total DNA, as described in the manufacturer’s protocol (Invitrogen, USA). Briefly, the cells in logarithmic growth phase were seeded on coverslips, incubated at 37° C for 1 h with 10 μM EdU, fixed with 3.7% formaldehyde, permeabilized by Triton X-100 (0.5%) treatment, stained with Alexa Fluor 488 and Hoechst 33342, and viewed in a luminescence microscope at wavelengths (excitation/emission, nm) 495/519 for Alexa Fluor 488 and 350/461 for Hoechst 33342.

### SDS-PAGE and western-blotting

The procedures were performed as described in [40]. 30 μg of cell lysate proteins were run on SDA-PAGE and electroblotted onto a PVDF membrane. After reaction with specific primary antibodies, the membrane was probed with HRP-conjugated secondary antibodies, developed in a ECL detection system (Amersham, England) and imaged on ChemiDoc (Bio-Rad; this device pertains to the equipment of “Human Proteome” Core Facility of the Institute of Biomedical Chemistry). Quantification of protein levels from western blot analysis was done using Image Lab software (Bio-Rad).

### Statistical analysis

Differences between the groups were assessed using Student’s *t*-test and considered significant at *p* < 0.05.

## References

[1] Hayflick L. Aging, longevity, and immortality *in vitro*. Exp Gerontol. 1992; 27:363–68. 10.1016/0531-5565(92)90066-9 1459211

[2] Mijit M, Caracciolo V, Melillo A, Amicarelli F, Giordano A. Role of p53 in the Regulation of Cellular Senescence. Biomolecules. 2020; 10:420. 10.3390/biom10030420 32182711 PMC7175209

[3] van Deursen JM. The role of senescent cells in ageing. Nature. 2014; 509:439–46. 10.1038/nature13193 24848057 PMC4214092

[4] Rodier F, Campisi J. Four faces of cellular senescence. J Cell Biol. 2011; 192:547–56. 10.1083/jcb.201009094 21321098 PMC3044123

[5] Hanahan D, Weinberg RA. Hallmarks of cancer: the next generation. Cell. 2011; 144:646–74. 10.1016/j.cell.2011.02.013 21376230

[6] Jun JI, Lau LF. The matricellular protein CCN1 induces fibroblast senescence and restricts fibrosis in cutaneous wound healing. Nat Cell Biol. 2010; 12:676–85. 10.1038/ncb2070 20526329 PMC2919364

[7] Blokland KE, Pouwels SD, Schuliga M, Knight DA, Burgess JK. Regulation of cellular senescence by extracellular matrix during chronic fibrotic diseases. Clin Sci (Lond). 2020; 134:2681–706. 10.1042/CS20190893 33084883 PMC7578566

[8] Gutiérrez-Fernández A, Soria-Valles C, Osorio FG, Gutiérrez-Abril J, Garabaya C, Aguirre A, Fueyo A, Fernández-García MS, Puente XS, López-Otín C. Loss of MT1-MMP causes cell senescence and nuclear defects which can be reversed by retinoic acid. EMBO J. 2015; 34:1875–88. 10.15252/embj.201490594 25991604 PMC4547893

[9] Hynes RO. Integrins: bidirectional, allosteric signaling machines. Cell. 2002; 110:673–87. 10.1016/s0092-8674(02)00971-6 12297042

[10] Takada Y, Ye X, Simon S. The integrins. Genome Biol. 2007; 8:215. 10.1186/gb-2007-8-5-215 17543136 PMC1929136

[11] Moreno-Layseca P, Icha J, Hamidi H, Ivaska J. Integrin trafficking in cells and tissues. Nat Cell Biol. 2019; 21:122–32. 10.1038/s41556-018-0223-z 30602723 PMC6597357

[12] Desgrosellier JS, Cheresh DA. Integrins in cancer: biological implications and therapeutic opportunities. Nat Rev Cancer. 2010; 10:9–22. 10.1038/nrc2748 20029421 PMC4383089

[13] Hamidi H, Ivaska J. Every step of the way: integrins in cancer progression and metastasis. Nat Rev Cancer. 2018; 18:533–48. 10.1038/s41568-018-0038-z 30002479 PMC6629548

[14] Cooper J, Giancotti FG. Integrin Signaling in Cancer: Mechanotransduction, Stemness, Epithelial Plasticity, and Therapeutic Resistance. Cancer Cell. 2019; 35:347–67. 10.1016/j.ccell.2019.01.007 30889378 PMC6684107

[15] Liu X, Yin D, Zhang Y, Zhao J, Zhang S, Miao J. Vascular endothelial cell senescence mediated by integrin beta4 *in vitro*. FEBS Lett. 2007; 581:5337–42. 10.1016/j.febslet.2007.10.027 17964297

[16] Jung SH, Lee M, Park HA, Lee HC, Kang D, Hwang HJ, Park C, Yu DM, Jung YR, Hong MN, Kim YN, Park HJ, Ko YG, Lee JS. Integrin α6β4-Src-AKT signaling induces cellular senescence by counteracting apoptosis in irradiated tumor cells and tissues. Cell Death Differ. 2019; 26:245–59. 10.1038/s41418-018-0114-7 29786073 PMC6329762

[17] Yuan L, Du X, Tang S, Wu S, Wang L, Xiang Y, Qu X, Liu H, Qin X, Liu C. ITGB4 deficiency induces senescence of airway epithelial cells through p53 activation. FEBS J. 2019; 286:1191–203. 10.1111/febs.14749 30636108

[18] Franovic A, Elliott KC, Seguin L, Camargo MF, Weis SM, Cheresh DA. Glioblastomas require integrin αvβ3/PAK4 signaling to escape senescence. Cancer Res. 2015; 75:4466–73. 10.1158/0008-5472.CAN-15-0988 26297735 PMC4631634

[19] Rapisarda V, Borghesan M, Miguela V, Encheva V, Snijders AP, Lujambio A, O’Loghlen A. Integrin Beta 3 Regulates Cellular Senescence by Activating the TGF-β Pathway. Cell Rep. 2017; 18:2480–93. 10.1016/j.celrep.2017.02.012 28273461 PMC5357738

[20] Huveneers S, Truong H, Danen HJ. Integrins: signaling, disease, and therapy. Int J Radiat Biol. 2007; 83:743–51. 10.1080/09553000701481808 17852562

[21] Xu Y, Li N, Xiang R, Sun P. Emerging roles of the p38 MAPK and PI3K/AKT/mTOR pathways in oncogene-induced senescence. Trends Biochem Sci. 2014; 39:268–76. 10.1016/j.tibs.2014.04.004 24818748 PMC4358807

[22] Sun Y, Liu WZ, Liu T, Feng X, Yang N, Zhou HF. Signaling pathway of MAPK/ERK in cell proliferation, differentiation, migration, senescence and apoptosis. J Recept Signal Transduct Res. 2015; 35:600–04. 10.3109/10799893.2015.1030412 26096166

[23] Cagnol S, Chambard JC. ERK and cell death: mechanisms of ERK-induced cell death--apoptosis, autophagy and senescence. FEBS J. 2010; 277:2–21. 10.1111/j.1742-4658.2009.07366.x 19843174

[24] Yan P, Zhu H, Yin L, Wang L, Xie P, Ye J, Jiang X, He X. Integrin αvβ6 Promotes Lung Cancer Proliferation and Metastasis through Upregulation of IL-8-Mediated MAPK/ERK Signaling. Transl Oncol. 2018; 11:619–27. 10.1016/j.tranon.2018.02.013 29573639 PMC6002349

[25] Ojalill M, Parikainen M, Rappu P, Aalto E, Jokinen J, Virtanen N, Siljamäki E, Heino J. Integrin α2β1 decelerates proliferation, but promotes survival and invasion of prostate cancer cells. Oncotarget. 2018; 9:32435–47. 10.18632/oncotarget.25945 30197754 PMC6126696

[26] Morozevich GE, Kozlova NI, Susova OY, Karalkin PA, Berman AE. Implication of α2β1 integrin in anoikis of MCF-7 human breast carcinoma cells. Biochemistry (Mosc). 2015; 80:97–103. 10.1134/S0006297915010113 25754044

[27] Pan HC, Jiang Q, Yu Y, Mei JP, Cui YK, Zhao WJ. Quercetin promotes cell apoptosis and inhibits the expression of MMP-9 and fibronectin via the AKT and ERK signalling pathways in human glioma cells. Neurochem Int. 2015; 80:60–71. 10.1016/j.neuint.2014.12.001 25481090

[28] Kozlova NI, Morozevich GE, Ushakova NA, Berman AE. Implication of integrin α2β1 in anoikis of SK-Mel-147 human melanoma cells: a non-canonical function of Akt protein kinase. Oncotarget. 2019; 10:1829–39. 10.18632/oncotarget.26746 30956761 PMC6443001

[29] Kozlova NI, Morozevich GE, Gevorkian NM, Berman AE. Implication of integrins α3β1 and α5β1 in invasion and anoikis of SK-Mel-147 human melanoma cells: non-canonical functions of protein kinase Akt. Aging (Albany NY). 2020; 12:24345–56. 10.18632/aging.202243 33260159 PMC7762463

[30] Kozlova NI, Morozevich GE, Ushakova NA, Berman AE. Implication of Integrin α2β1 in Proliferation and Invasion of Human Breast Carcinoma and Melanoma Cells: Noncanonical Function of Akt Protein Kinase. Biochemistry (Mosc). 2018; 83:738–45. 10.1134/S0006297918060111 30195330

[31] Rayess H, Wang MB, Srivatsan ES. Cellular senescence and tumor suppressor gene p16. Int J Cancer. 2012; 130:1715–25. 10.1002/ijc.27316 22025288 PMC3288293

[32] Dolan DW, Zupanic A, Nelson G, Hall P, Miwa S, Kirkwood TB, Shanley DP. Integrated Stochastic Model of DNA Damage Repair by Non-homologous End Joining and p53/p21-Mediated Early Senescence Signalling. PLoS Comput Biol. 2015; 11:e1004246. 10.1371/journal.pcbi.1004246 26020242 PMC4447392

[33] Spallarossa P, Altieri P, Aloi C, Garibaldi S, Barisione C, Ghigliotti G, Fugazza G, Barsotti A, Brunelli C. Doxorubicin induces senescence or apoptosis in rat neonatal cardiomyocytes by regulating the expression levels of the telomere binding factors 1 and 2. Am J Physiol Heart Circ Physiol. 2009; 297:H2169–81. 10.1152/ajpheart.00068.2009 19801496

[34] Weichhart T. mTOR as Regulator of Lifespan, Aging, and Cellular Senescence: A Mini-Review. Gerontology. 2018; 64:127–34. 10.1159/000484629 29190625 PMC6089343

[35] Revathidevi S, Munirajan AK. Akt in cancer: Mediator and more. Semin Cancer Biol. 2019; 59:80–91. 10.1016/j.semcancer.2019.06.002 31173856

[36] Han X, Liu D, Zhang Y, Li Y, Lu W, Chen J, Songyang Z. Akt regulates TPP1 homodimerization and telomere protection. Aging Cell. 2013; 12:1091–99. 10.1111/acel.12137 23862686 PMC4051222

[37] Chung J, Khadka P, Chung IK. Nuclear import of hTERT requires a bipartite nuclear localization signal and Akt-mediated phosphorylation. J Cell Sci. 2012; 125:2684–97. 10.1242/jcs.099267 22366458

[38] Thaler S, Hähnel PS, Schad A, Dammann R, Schuler M. RASSF1A mediates p21Cip1/Waf1-dependent cell cycle arrest and senescence through modulation of the Raf-MEK-ERK pathway and inhibition of Akt. Cancer Res. 2009; 69:1748–57. 10.1158/0008-5472.CAN-08-1377 19223555

[39] Jee HJ, Kim HJ, Kim AJ, Bae YS, Bae SS, Yun J. UV light induces premature senescence in Akt1-null mouse embryonic fibroblasts by increasing intracellular levels of ROS. Biochem Biophys Res Commun. 2009; 383:358–62. 10.1016/j.bbrc.2009.04.017 19364500

[40] Yang J, Nie J, Ma X, Wei Y, Peng Y, Wei X. Targeting PI3K in cancer: mechanisms and advances in clinical trials. Mol Cancer. 2019; 18:26. 10.1186/s12943-019-0954-x 30782187 PMC6379961

[41] Morozevich GE, Kozlova NI, Ushakova NA, Preobrazhenskaya ME, Berman AE. Integrin α5β1 simultaneously controls EGFR-dependent proliferation and Akt-dependent pro-survival signaling in epidermoid carcinoma cells. Aging (Albany NY). 2012; 4:368–74. 10.18632/aging.100457 22626691 PMC3384437

[42] Morozevich G, Kozlova N, Cheglakov I, Ushakova N, Berman A. Integrin alpha5beta1 controls invasion of human breast carcinoma cells by direct and indirect modulation of MMP-2 collagenase activity. Cell Cycle. 2009; 8:2219–25. 10.4161/cc.8.14.8980 19617714

